# Pneumococcal DNA is not detectable in the blood of healthy carrier children by real-time PCR targeting the *lytA* gene

**DOI:** 10.1099/jmm.0.028357-0

**Published:** 2011-06

**Authors:** Chiara Azzari, Martina Cortimiglia, Maria Moriondo, Clementina Canessa, Francesca Lippi, Federica Ghiori, Laura Becciolini, Maurizio de Martino, Massimo Resti

**Affiliations:** Department of Paediatrics, Anna Meyer Children’s University Hospital, Florence, Italy

## Abstract

The diagnosis of invasive pneumococcal disease (IPD) is currently based on culture methods, which lack sensitivity, especially after antibiotic therapy. Molecular methods have improved sensitivity and do not require viable bacteria; however, their use is complicated by reports of low specificity with some assays. The present study investigated the specificity of a real-time PCR targeting *lytA* for the detection of IPD. A group of 147 healthy children, aged 6 months to 16 years (mean 6.4 years, median 4.9 years, interquartile range 6.4 years), who were in hospital for routine examinations, were tested for pneumococcal carrier status and for the presence of detectable pneumococcal DNA in their blood by real-time PCR targeting the pneumococcal *lytA* gene. In addition, 35 culture-positive biological samples were analysed. Urine was examined for the presence of pneumococcal DNA and C-polysaccharide antigen. Carriage was detected in 77 of the 147 subjects (52.4 %); however, regardless of carrier status, none of the subjects had a positive result from blood. Analysis of the culture-positive biological samples yielded positive results in 100 % (15/15) of cerebrospinal fluid samples and 95 % (19/20) of blood samples. All urine samples from healthy carriers were negative for DNA, whilst antigenuria was detected in 44/77 carriers (57.1 %). In conclusion, real-time PCR is both sensitive and specific and can be a useful tool in the routine diagnosis of IPD. Its sensitivity, which surpasses that of other methods for this purpose, does not come at the cost of reduced specificity.

## Introduction

*Streptococcus pneumoniae* is the leading cause of invasive bacterial infections in children. Worldwide, an estimated 10.6 million children below the age of 5 years present with pneumococcal disease every year. Other at-risk populations include the elderly and immunosuppressed. Overall, more than 1.6 million deaths per year are attributable to such infections (Black *et al.*, 2003; WHO, 2007). Establishing a microbiological diagnosis is important for proper treatment and epidemiological analysis.

Culture has been considered the gold standard for diagnosing invasive pneumococcal disease (IPD). However, this method suffers from a lack of sensitivity, especially when used after antibiotic therapy has been started to treat the infection (Azzari *et al.*, 2010; Resti *et al.*, 2009) or when small volumes are available, as is common in paediatric practice (Isaacman *et al.*, 1996). An immunochromatographic method to detect bacterial cell-wall C polysaccharide antigen excreted in urine is sensitive but does not appear to distinguish between colonization and invasive disease. Potentially, these obstacles could be overcome by the use of quantitative PCR.

Recently, it has been demonstrated that molecular methods for detecting bacterial DNA in blood or other normally sterile body fluids can be used for the efficient diagnosis and serotyping of IPD (Antonio *et al.*, 2009; Azzari *et al.*, 2008, 2010; Blaschke *et al.*, 2010; Carvalho *et al.*, 2010; Chiba *et al.*, 2009; Corless *et al.*, 2001; Saha *et al.*, 2008; Strachan *et al.*, 2011; Van Gastel *et al.*, 2007). These methods do not require viable bacteria, need only small sample volumes and appear to be more sensitive than culture methods, making them potentially useful tools for the diagnosis and serotyping of pneumococcal pneumonia (Resti *et al.*, 2010), even in conjunction with antibiotic treatment (Resti *et al.*, 2009). A large number of clinical diagnostic laboratories around the world now use molecular methods as part of their routine testing. However, whenever a test appears to be more sensitive than those previously considered as gold standards, it is important to determine whether this increase in sensitivity is accompanied by a reduction in specificity and hence an increase in the rate of false-positives. For example, tests performed in the past using end-point PCR and primers targeting part of the streptococcal pneumolysin (*ply*) gene sequence reported finding positive results in the blood of healthy subjects even in the absence of *S. pneumoniae* colonization (Dagan *et al.*, 1998; Murdoch *et al.*, 2003).

The aim of the present study was to determine the frequency of positive PCR results in the blood of healthy carriers and non-carriers (i.e. false-positives) when assayed with a sensitive real-time PCR employing reagents to detect the pneumococcal autolysin (*lytA*) gene sequence, which is reported to be more specific than other genes such as* ply* (Abdeldaim *et al.*, 2010; Carvalho *et al.*, 2007).

## Methods

### 

#### Subjects.

The study included 147 healthy children between the ages of 6 months and 16 years (median 4.9 years, interquartile range 6.4 years), who were present at the Anna Meyer Children’s Hospital, Florence, Italy, for routine examinations related to allergies or coeliac disease. None of the healthy subjects had taken antibiotics or received anti-pneumococcal vaccination for at least 1 month prior to the examination. Culture-positive biological samples of blood (20 samples) and cerebrospinal fluid (15 samples) were also tested. Moreover, real-time PCR was performed on 15 urine samples from patients with invasive pneumococcal infection (meningitis or pneumonia) whose blood samples were positive by real-time PCR.

#### PCR conditions.

The presence of *S. pneumoniae* DNA in blood, urine or nasal swabs was evaluated by real-time PCR as described previously (Azzari *et al.*, 2010). Primers and probes within the previously published *lytA* gene sequence (Carvalho *et al.*, 2007) were used in PCR amplifications at a final concentration of 400 nM for the primers and 400 nM for the probe, which was labelled with 6-carboxy-4′,5′-dichloro-2′,7′-dimethoxyfluorescein (JOE). A negative control (no template) and a positive control were included in every PCR run. Total DNA was extracted from 200 µl blood or urine using a QIAmp DNeasy Blood & Tissue kit (Qiagen), according to the manufacturer’s instructions. Nasal swabs were placed in 1 ml PBS and mixed by vortexing for 1 min, and DNA was extracted from a 200 µl sample. Real-time PCR was performed with an ABI 7000 sequence detection system (Applied Biosystems) using the following cycling parameters: 95 °C for 10 min, followed by 45 cycles of 95 °C for 15 s and 60 °C for 1 min. Cycle threshold (*C*_t_) values were used to identify amplification of the target sequence and to estimate bacterial load. Samples were assumed to be negative if no increase in fluorescence signal was observed after 45 cycles. All reactions were performed in triplicate.

For quantitative analysis of carriage density, the amount of bacterial DNA was obtained by direct extrapolation of the *C*_t_ values to the amount of DNA (in copies ml^−1^) as read from the concentration versus *C*_t_ standard curve, as described previously (Rello *et al.*, 2009; Resti *et al.*, 2010).

*S. pneumoniae* bacterial cell-wall antigens were detected using a C polysaccharide antigen NOW Urinary Antigen Assay (Binax) according to the manufacturer’s instructions.

#### Power of the study.

Very few data are available on PCR positivity for *S. pneumoniae* in the blood of healthy children (Dagan *et al.*, 1998; Murdoch *et al.*, 2003).

A rate of false positivity in the diagnosis of invasive pneumococcal infection below 0.7 % was considered acceptable in the clinical setting, being one-quarter of the false-positive samples obtained with culture methods, which, in a paediatric setting, appears to be around 2.8 % (Sard *et al.*, 2006). For that reason, as we hypothesized that we would not find a single positive with our method, the study was designed to include at least 142 healthy children, in the hope of finding at least one positive result that would allow quantification of the false-positive rate. For comparison of our method (real-time PCR of *lytA*) with previous reports evaluating the presence of pneumococcal DNA in healthy children (nested end-point PCR of *ply*), assuming a carriage rate of 50 % for the paediatric population studied, a sample size of only 80 subjects would be sufficient to detect a statistically significant difference.

## Results and Discussion

The low sensitivity of methods that require culture for the detection of pneumococcal disease may be due in part to their sensitivity to antibiotic therapy. PCR is a more sensitive assay (Antonio *et al.*, 2009; Azzari *et al.*, 2008, 2010; Corless *et al.*, 2001; Saha *et al.*, 2008; Van Gastel *et al.*, 2007); however, the question of its specificity has been raised by the reported findings of positive PCR results from the blood of healthy subjects (Dagan *et al.*, 1998; Murdoch *et al.*, 2003). Both of these studies used sequences from the pneumococcal *ply* gene as the target for PCR. Recently, the specificity of the *ply* gene as a target for PCR-based detection of IPD has been questioned (Abdeldaim *et al.*, 2009; Neeleman *et al.*, 2004). When *ply* is used for diagnosis of pneumococcal infection on bronchoalveolar lavage or sputum samples, the presence in such samples of other bacteria such as *Streptococcus mitis* or *Streptococcus oralis* can be the cause of false-positive results, as these bacteria are known to contain similar sequences (Abdeldaim *et al.*, 2009). However, it is difficult to imagine that a similar mechanism might be the cause of positivity for *ply* sequences in the blood of healthy subjects, and technical issues should also be considered. In fact, the studies that found positivity for *ply* sequences in the blood of healthy subjects used a nested end-point PCR, which is known to give a higher rate of false-positive results because it requires greater manipulation of the samples and amplicons. Recently, the *lytA* gene sequence has been suggested as more specific for the diagnosis of pneumococcal disease (Abdeldaim *et al.*, 2010; Carvalho *et al.*, 2007) and real-time PCR appears to be a more sensitive and specific tool for microbiological analysis. Therefore, we undertook the present study to determine the false-positive rate using real-time PCR with the pneumococcal *lytA* gene as the target.

We cannot exclude the possibility that carriage densities in the previous studies were higher than in our patients, thus affecting the passage of bacteria to the blood. However, the results of our study seem to contradict this hypothesis; in fact, the carriage density in the 77 carrier children in our study was in some subjects very high: values ranged between 1.6 and 5.2 log copies ml^−1^, with a mean value of 2.4 log copies ml^−1^ and an interquartile range of 2.2–3.7 log copies ml^−1^.

### Carrier status

**Fig. 1.**
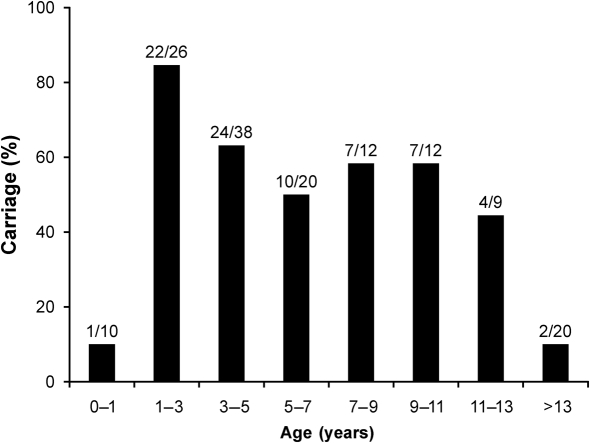
*S. pneumoniae* carrier status according to age, as determined by real-time PCR analysis of nasal swabs.

In total, 77 of the 147 subjects (52.4 %) were positive for pneumococcal colonization of the pharynx using our real-time PCR method on nasal swabs. This is consistent with the expected 50 % carriage rate for this population that we used in our power calculation. The age distribution of carrier status is presented in Fig. 1. There were few carriers among children under the age of 1 year and among children over the age of 13 years. This reflects the typical pattern of low carriage rates for very young children, followed by increasing rates that peak between 1 and 2 years of age and then decline, rates being much lower in adults (Antonio *et al.*, 2009; Azzari & Resti, 2008; Leino *et al.*, 2001; Regev-Yochay *et al.*, 2004).

### Pneumococcal DNA in blood

Regardless of carrier status, we did not find positive results with PCR in the blood of any of the 147 subjects tested. The incidence of false-positive results appeared, therefore, to be equal to zero or at least less than 1/147 (<0.7 %) for children under the age of 16 years. PCR analysis of the culture-positive biological samples yielded positive results in 100 % (15/15) of cerebrospinal fluid samples and 95 % (19/20) of blood samples, with an overall sensitivity of 97 % (34/35). This is in agreement with our findings in a large population of children with pneumonia (Resti *et al.*, 2010), where among the 292 patients who were tested with both culture and real-time PCR methods, *S. pneumoniae* was detected by one of the two methods in 47 patients, 45 of whom (96 %) were positive by real-time PCR with a sensitivity of real-time PCR that was four times higher than that of culture (Resti *et al.*, 2010).

### Antigenuria

We found that, despite having negative PCR results for pneumococcal DNA in the blood, 44 of the 77 carriers (57.1 %) had positive results for antigen in the urine, whilst the 70 non-carriers were all negative. This finding is consistent with previous reports of positive tests in a large percentage of children (Charkaluk *et al.*, 2006; Domínguez *et al.*, 2003; Dowell *et al.*, 2001). In an attempt to understand this phenomenon, we examined urine samples for the presence of bacterial DNA and found none, indicating that colonization of the urinary tract was unlikely. We examined whether a correlation existed between the bacterial load colonizing the pharynx, as estimated by quantitative real-time PCR, and positivity for antigen in the urine, but did not find a significant correlation (*P* = 0.41). Moreover, there was no strong correlation between antigen in the urine and age (*P* = 0.09).

The absence of pneumococcal DNA in the blood and urine argues against disseminated infection as the source of antigen. We speculate that the antigenuria results from ingestion of colonized nasal secretions, followed by absorption of C-polysaccharide antigen from the digestive tract and excretion by the kidneys. Our findings serve to underline that, whilst useful for testing in adults where the carriage rate is low (Smith *et al.*, 2009), tests for C-polysaccharide antigen in urine are not useful in children because a positive result can be due to either colonization or IPD. With regard to the presence of pneumococcal DNA in the urine of IPD patients, we demonstrated that only 2/15 blood-positive IPD patients (13.3 %) also had positive urine samples. PCR performed on urine samples thus seems less sensitive than PCR or cultures performed on blood in patients with IPD. Larger studies designed to compare PCR sensitivity in different biological fluids are needed to confirm this point.

Real-time PCR assays of normally sterile body fluids, on the other hand, are specific for invasive disease in children and are more sensitive than culture methods. Using highly sensitive real-time PCR avoids the use of nested PCR assays, which are associated with lower specificity (Avni *et al.*, 2010) and present more possibilities for introducing method-based false-positives from contamination.

One limitation of the present study was that it was only performed on children with intact immune responses. Future studies will need to investigate the other two major at-risk populations – adults over the age of 65 and immunosuppressed subjects (Black *et al.*, 2003) – to determine whether the method is also applicable to these populations.

In conclusion, real-time PCR detecting the *lytA* gene sequence is both sensitive and specific and can be a useful tool in the routine diagnosis of IPD. Its sensitivity, which surpasses that of any other method for this purpose, does not come at the cost of reduced specificity.
